# Oxytocin accelerates tight junction formation and impairs cellular migration in 3D spheroids: evidence from Gapmer-induced exon skipping

**DOI:** 10.3389/fncel.2022.1000538

**Published:** 2022-10-03

**Authors:** Benjamin Jurek, Lucia Denk, Nicole Schäfer, Mohammad Saied Salehi, Sareh Pandamooz, Silke Haerteis

**Affiliations:** ^1^Institute for Molecular and Cellular Anatomy, University of Regensburg, Regensburg, Germany; ^2^Research Group Neurobiology of Stress Resilience, Max Planck Institute of Psychiatry, Munich, Germany; ^3^Experimental Orthopaedics, Centre for Medical Biotechnology (ZMB), Bio Park 1, University of Regensburg, Regensburg, Germany; ^4^Clinical Neurology Research Center, Shiraz University of Medical Science, Shiraz, Iran; ^5^Stem Cells Technology Research Center, Shiraz University of Medical Sciences, Shiraz, Iran

**Keywords:** oxytocin, Gapmer-induced exon skipping, spheroid, tight junction, intracellular signaling

## Abstract

Oxytocin (OXT) is a neuropeptide that has been associated with neurological diseases like autism, a strong regulating activity on anxiety and stress-related behavior, physiological effects during pregnancy and parenting, and various cellular effects in neoplastic tissue. In this study, we aimed to unravel the underlying mechanism that OXT employs to regulate cell-cell contacts, spheroid formation, and cellular migration in a 3D culture model of human MLS-402 cells. We have generated a labeled OXT receptor (OXTR) overexpressing cell line cultivated in spheroids that were treated with the OXTR agonists OXT, Atosiban, and Thr^4^-Gly^7^-oxytocin (TGOT); with or without a pre-treatment of antisense oligos (Gapmers) that induce exon skipping in the human OXTR gene. This exon skipping leads to the exclusion of exon 4 and therefore a receptor that lost its intracellular G-protein-binding domain. Sensitive digital PCR (dPCR) provided us with the means to differentiate between wild type and truncated OXTR in our cellular model. OXTR truncation differentially activated intracellular signaling cascades related to cell-cell attachment and proliferation like Akt, ERK1/2-RSK1/2, HSP27, STAT1/5, and CREB, as assessed by a Kinase Profiler Assay. Digital and transmission electron microscopy revealed increased tight junction formation and well-organized cellular protrusions into an enlarged extracellular space after OXT treatment, resulting in increased cellular survival. In summary, OXT decreases cellular migration but increases cell-cell contacts and therefore improves nutrient supply. These data reveal a novel cellular effect of OXT that might have implications for degenerating CNS diseases and tumor formation in various tissues.

## Introduction

The neuropeptide oxytocin (OXT) has been implicated in many behavioral and physiological processes, like the formation of parent-offspring bonding, labor and milk-letdown, social interaction, general anxiety, and stress-coping (Jurek and Neumann, 2018). A subset of patients suffering from central nervous system (CNS)-related disorders like autism-spectrum disorder, general anxiety, or schizophrenia could benefit from intranasal applications of OXT (Hurlemann, 2017), potentially by modulating the social salience network (Shamay-Tsoory and Abu-Akel, 2016; Jurek and Meyer, 2020), but with a yet-unknown cellular/molecular mechanism. On a cellular level, OXT induces mitogenic and calcium-related signaling cascades *via* the direct binding to its OXT receptor (OXTR; Busnelli and Chini, 2018), the vasopressin receptors (V1a, V1b, V2; Chini and Manning, 2007), or the TrpV1 channel (Nersesyan et al., 2017). The agonist Thr^4^-Gly^7^-oxytocin (TGOT) is specific for the OXTR with a very low affinity for the V1a/b/V2 receptors, thus it can serve as an internal control for OXTR activation (Busnelli et al., 2013). Both, OXT and TGOT activate second messenger signaling either by inhibitory Gα_i_, Gα_o_, or bystimulatory Gα_q_-protein coupling to the OXTR, depending on the ligand concentration (Gimpl and Fahrenholz, 2001). Biased agonists, such as Atosiban, bind the OXTR but selectively activate the inhibitory Gα_i_-protein-coupled pathways (Reversi et al., 2005). Upon OXTR activation and in addition to G-protein signaling, β-arrestin also binds the OXTR at the intracellular loops of its transmembrane domains and induces G-protein-independent signaling cascades (Grotegut et al., 2011; Passoni et al., 2016). Signaling cascades that have been linked to the activated OXTR encompass the MAPK pathways (Jurek et al., 2012), calcium-related cascades (Meyer et al., 2022), and the activation of transcription factors like CREB or MEF2A (Winter et al., 2021). Once activated, the OXTR undergoes desensitization, internalization, and either degradation or recycling back to the cell surface (Conti et al., 2009; Passoni et al., 2016). The stability of the OXTR, its ligand-affinity, and its subsequent signaling response depend on hardly manageable factors, like the genetic variant (Meyer et al., 2022), the membrane cholesterol content (Reversi et al., 2006a; Muth et al., 2011), and its location in caveolin-enriched domains in the cellular membrane, which affects the temporal pattern of mitogenic signaling (Rimoldi et al., 2003).

As a consequence of the promiscuous coupling to inhibitory or stimulatory G-proteins, OXT inhibits or stimulates cellular proliferation under physiological and neoplastic conditions (Cassoni et al., 1994, 1996, 2000, 2001, 2004; Reversi et al., 2006b). The cell types in which OXT induces cell proliferation range from primary astrocytes, epithelial adenocarcinoma MCF7 cells, human osteoblast-like cells, small cell lung carcinoma cells, to human dermal microvascular endothelial cells, or endothelial sarcomatous cell lines. Cell types in which OXT inhibits cell proliferation include neuroblastoma cells (IMR32, SH-N-SH, SH-SY5Y), epithelial cells (HUVEC, HAEC, HPAEC), adenocarcinoma of the endometrium, breast cancer cells (T47D, MDA-MB231), ovarian carcinoma (SKOV3), endometrial carcinoma (Colo684, A-MEC, HEC1A), prostate cancer cells (DU145), and osteosarcoma cells (MG-63, U2OS; for an overview of cell-types see Reversi et al., 2006b and references therein). In addition to proliferation, OXT stimulates cellular migration in human endothelial and prostate cancer cells (Cattaneo et al., 2008; Zhong et al., 2010), but inhibits migration in head and neck squamous cell carcinoma cells. Moreover, the same study also found OXT-induced size-reduction and inhibition of 3D-spheroid formation (Kim et al., 2017). In contrast, we have recently shown that OXT increases the spheroid size in rat hypothalamic neurons (Salehi et al., 2021). The spheroid formation, proliferation, and cellular migration are being orchestrated by the cells’ ability to perform cell-cell contacts or cell-matrix interactions (Atat et al., 2022), cellular characteristics that are important hallmarks in degenerative disorders of the CNS or tumor formation in the periphery (Stadler et al., 2018; Atat et al., 2022). In this study, we have made use of the liposarcoma cell line MLS-402, which was virally transduced to overexpress a fluorescently labeled OXTR. MLS-402 cells form spheroids and serve as an ideal model system for neoplastic endothelial cells, but also to study general intracellular cascades and cell-cell interactions.

The contrasting effects on proliferation and spheroid formation, which are dependent on above-mentioned highly variable factors, render OXT a suboptimal treatment option for any medical regimen; however, detailed knowledge of the underlying mechanism that allows both pro- and anti-proliferative effects, might provide us with a tool to enforce any desired cellular response. Consequently, the aim of this study is to characterize the intracellular response to OXTR ligands with novel approaches, such as Gapmer-induced exon skipping, to gain a better understanding of the decisive factors that regulate OXT-induced cellular processes.

Antisense oligo (Gapmer)-induced exon skipping is an innovative technique that relies on the blockade of exonic splice enhancer (ESE) and splice acceptor sites by modified oligos (Li et al., 2018; Flynn et al., 2021). Those so-called Gapmers are antisense oligos whose backbones are chemically modified for increased stability, less toxicity, and the ability to enter cells *via* gymnosis (Winter et al., 2021). In this study, we have made use of Gapmers that were designed to bind to ESEs within exon 4 of the *OXTR*, so that exon 4 will be skipped from the mature mRNA. If exon 4 is missing in the mature mRNA due to exon skipping, the resulting receptor lacks the intracellular domain and therefore the ability to activate intracellular signaling cascades *via* G-proteins.

The overall expression level of the OXTR protein in any given cell is generally very low, with some physiological exceptions such as the gestational myometrium (Akerlund et al., 1999). This fact can be attributed to the general toxicity of overexpressed GPCRs in eukaryotic cells (Rajagopal and Shenoy, 2018). The method of choice to differentiate between wild type and mutated OXTR is digital PCR, a novel and more sensitive form of quantitative PCR that is able to reliably detect the wild type and mutated OXTR in absolute values, without the need for a standard curve (Huggett, 2020). We designed the *OXTR*-detection probes to specifically bind the exon 2–3 boundary, or to bind within exon 4. This approach allows us to differentiate between a wild type and a truncated receptor that is lacking the exon 4 and therefore its intracellular domain.

In summary, this study employed a novel approach to investigate the molecular mechanism underlying the diverse effects of OXT on cell-cell contacts, proliferation, and migration in a 3D spheroid environment, which provides the basis for a better understanding of degenerative diseases and cancer.

## Material and Methods

### Cell culture

Human MLS-402 (liposarcoma cells kindly provided by Prof. Dr. Steffen Eisenhardt, University Hospital Freiburg, and their derivate subclone overexpressing EGFP-OXTR-mCherry (MLS-G2^OXTR^) and the control subclone MLS-402^EGFP-mCherry^ were cultured in RPMI-1640 medium, with L-glutamine and sodium bicarbonate (#R8758, Sigma Aldrich, Darmstadt, Germany), supplemented with 10% fetal bovine serum (#S1810, Biowest, Nuaillé, France) at 37°C and 5% CO_2_ until 80% confluency. Cells were kept under antibiotic-free sterile conditions and tested for mycoplasmic contamination on a regular basis. Passaging was performed at least once a week by gentle trypsinization. Cell counts were recorded and compared between MLS-402 and MLS-G2^OXTR^ cell lines to extrapolate proliferation rates.

### Viral transduction and monoclonal cell line creation

EGFP and mCherry labeled human OXTR under the control of a CMV early enhancer/chicken β-actin promoter was transduced into MLS-402 cells using a lentiviral system (pLV[Exp]-Hygro-CBh > EGFP/hOXTR[NM_001354654.1]/ mCherry, VectorBuilder, Berlin, Germany). A control virus (pLV[Exp]-Hygro-CBh > EGFP/mCherry) was used to assess the side effects of transduction and EGF/mCherry expression. 10 MOI of viral particles were used, and 250 μg/ml hygromycin (Sigma Aldrich, H3274) for 3 days to select successfully transduced cells. Vital, proliferating cells were singled out in 96-well plate, and resulting colonies were cryo-preserved until further analysis per digital PCR.

### RNA isolation, cDNA synthesis, and digital PCR (dPCR)

Cells were lysed using 1 ml Ambion TRIzol (#15596018, Invitrogen, Carlsbad, USA) per 6-well according to the manufacturer’s protocol, the resulting RNA phase was washed and DNA digested using the RNeasy Mini Kit (#74104 and #79254, Qiagen, Hilden Germany), 1 μg/μl of cDNA was synthesized with the iScript cDNA synthesis Kit (#1708890, BioRad, Feldkirchen, Germany), the resulting cDNA was used undiluted for dPCR-based detection of the OXTR transcript with a Qiacuity dPCR cycler (Qiagen) in an 8.5 k 24-well Qiacuity Nanoplate and QuantiNova LNA probe PCR assays (see Table 1). Homo sapiens ACTB was used as a reference gene to control for even cDNA content. Cycling conditions were as follows: 2 min at 95°C heat activation, 15 s 95°C denaturation, 30 s 60°C annealing/extension for 40 cycles. Imaging was set to 500 ms exposure and gain of 20.

**Table 1:** Technical details of the QuantiNova LNA Probe PCR assays (Qiagen).

**Probe name**	**Amplicon start**	**Exon**	**Amplicon length**	**Ensembl Gene ID**
HS_OXTR_2484965	423	2–3 boundary	80	ENSG00000180914
HS_OXTR_2484969	1,395	3–4 boundary	228	ENSG00000180914
HS_OXTR_170144	2,203	4	167	ENSG00000180914
HS_ACTB_2476226	433	3–4 boundary	85	ENSG00000075624

### Spheroid cultivation

MLS-G2 cells were thawed and sub-cultured for at least two times under Hygromycin selection medium (50 μg/ml). Then, 1 × 10^5^ cells were seeded in a 96-well plate sterile-coated with 1.5% agarose for 24 h or 3 days in 200 μl normal growth medium with or without immediate addition of OXTR ligands and Gapmers.

### Gapmer transfection and OXTR ligand stimulation

After seeding cells in agarose coated 96-well plates, negative control LNA Gapmer A (referred to as n.c., #339999) or a 1:1:1 mixture of three custom designed LNA Gapmers (see Table 2) that bind exonic splice enhancer and acceptor sites are pre-mixed with 1 μl Lipofectamine RNAiMax (Invitrogen) per 200 μl medium and gently added to the forming spheroids. After Gapmer transfection, OXTR ligands OXT (#4016373, Bachem, Bubendorf, Switzerland), Atosiban (#6332, Tocris, Wiesbaden, Germany), and TGOT (#4013837, Bachem) are added at 100 nM or 1 μM for 24 h.

**Table 2:** Sequence and binding site of OXTR specific Gapmers and negative control (referred to as n.c. in the figures) Gapmers.

**Product name**	**Target**	**Product sequence 5’–3’**
OXTR-ESE1-V1	Exonic splice enhancer site Exon 4, hOXTR	/Biosg/A*C*A*G*G*A*A*G*C*G*C*T*G*C*A*C*G*A*G*T*T
OXTR-ESE2-V1	Exonic splice enhancer site, Exon 4, hOXTR	/Biosg/A*G*C*A*G*C*T*C*C*T*C*T*G*G*C*T*G*G*A
OXTR-E4ACC-V1	Exonic splice acceptor site, Exon 4, hOXTR	/Biosg/T*G*A*T*G*A*A*G*G*C*C*G*A*G*G*C*T*G*A*G
Negative control A	-	/Biosg/A*C*G*T*C*T*A*T*A*C*G*C*C*C*A

### Spheroid volume/area/sphericity assay

After 24 h of Gapmer and ligand stimulation, spheroids are imaged on a Leica DM IL LED microscope and spheroid area is assessed by means of Fiji ImageJ (1.53f51, NIH, USA). Spheroid volume is calculated by the formula 4/3π [(D1 + D2)/4]3, with D1 and D2 being the diameters. Spheroid volume was also recorded on the Keyence VHX7000 digital microscope using the 3D Panorama function. Sphericity was recorded using Fijis “Analyze particles” function with a cut-off of 0.7 being “spherical” and below 0.7 being “aspherical”. Spheroids with centric holes (donut-shape) are also classified as “aspherical”.

### Cellular migration assay

Twenty-four hours after Gapmer transfection and ligand stimulation, spheroids are transferred with their stimulation medium to a standard cell culture flat bottom 96-well plate. The spheroids are allowed to settle for 1 h and imaged as time point 0 on the Leica DM IL LED microscope. After 12 h and 84 h the spheroids are imaged again with identical microscope settings. Spheroids are cropped from time point 0 images and overlayed on time point 84 h to visualize the original spheroid size. Original spheroid size and the size of the migrated cell layer are put into ratio as % increase of migration area. As some of the spheroids are close to one well wall and consequently can only migrate asymmetrically, we assessed the 10 longest distances of cells traveled from the spheroid and recorded that as migration distance.

### Cell viability assay

To determine cell viability after 24 h of 100 nM OXT or TGOT, cells were seeded in 6-well plates at 1.5 × 10^6^ cells in normal growth medium. Cell viability was determined by means of the Promega CellTiter-Glo^TM^ cell viability assay according to the manufacturer’s instructions and analyzed *via* GloMax plate reader system.

### Nucleus size and count in spheroids

Gapmer- and ligand-treated spheroids were fixated using 4% paraformaldehyde for 20 min and nuclei were stained with Hoechst 333342 for nucleus count and size determination using the blue channel, auto threshold, and the “analyze particles” function in Fiji. The same ROI was used between different images, and all images were acquired with identical settings.

#### Live cell imaging

Live cell imaging was performed on an inverted Zeiss Axiovert 200 M microscope equipped with a heating element to retain 37°C. Cells were cultured on glass cover slides in a 6-well plate under normal growth medium conditions for 24 h. Cover slips were removed and placed inverted on a glass microscope slide (Menzel, Braunschweig Germany) with PBS. Images were processed and analyzed by Fiji ImageJ.

#### Transmission electron microscopy (TEM) imaging

For electron microscopy, the untreated (VEH) and treated (100 nM OXT) spheroids were primarily fixated in 2% glutaraldehyde (Serva, Heidelberg, Germany) buffered with 100 mM sodium cacodylate, pH 7.4 for 24 h at 4°C. For better handling during the following process, the spheroids were coated with 4% low melting agarose in 100 mM sodium cacodylate. After the addition of 1% osmium tetroxide (Science Services, München, Germany) for a secondary fixation step and 0.5% uranyl acetate for optimal contrast, the spheroids were dehydrated in a graded series of ethanol and embedded in two perpendicular orientations (diagonal and longitudinal) in Epon (Fluka, Taufkirchen, Germany), which was polymerized at 60°C for 3 days.

The ultrathin sections of the embedded spheroids were cut with a diamond knife (Diatome, Nidau, Switzerland) on an ultramicrotome EM UC7 (Leica, Wetzlar, Germany) and then collected on a slot grid coated with 1.5% Pioloform^®^ (Polyvinyl butyral), solved in chloroform (Plano, Wetzlar, Germany). The electron micrographs were taken at 80 kV on an EM 902 transmission electron microscope (Zeiss, Oberkochen, Germany; Minuth and Denk, 2015).

The captured TEM images were background subtracted (30 pixel rolling ball), smoothed, and distortions were corrected using the “distortion correction” plugin in Fiji/ImageJ (Kaynig et al., 2010) with a lambda of 9.51, and stitched using the MosaicJ plugin.

#### Correlative light electron microscopy (CLEM)

MLS-G2 cells were seeded on 35 mm glass bottom culture dishes with gridded coverslips (MatTek, P35G-1.5-14-CGRD), fixated in 4% paraformaldehyde in 100 mM cacodylate buffer, and imaged for OXTR-EGFP fluorescence at a Zeiss Axiovert 200 MOT. After imaging, the cells were post-fixated with 2% glutaraldehyde in 100 mM cacodylate buffer and treated as described for TEM imaging until alcohol dehydration. Lastly, acetone was used to disconnect the glass cover slip from the cell culture dish, and cells were embedded in 1:1 acetone/Epon for 5 min, and pure Epon for 1.5 h at 30°C. Final polymerization was achieved by incubation for 2 days at 60°C. Images were pre-processed identical to TEM imaging and imaged with the underlying grid for orientation. Fluorescent and TEM images were overlayed by means of the ec-CLEM plugin in ICY Bioimage Analysis software platform.

#### Digital 3D microscopy

A VHX7000 digital microscope at 200× magnification was used to assess the volume and 3D-surface characteristics of 4% PFA-fixated spheroids covered by 50–100 μl PBS on a black imaging surface, which guarantees that spheroids retain their native shape but are accessible for 3D imaging, undisturbed by surrounding well-walls. The black and flat imaging surface is essential for setting a baseline and subsequent correct volume determination.

#### Machine learning based image segmentation

TEM micrographs were segmented into four classes: “extracellular space”, “cytoplasm”, “organelles”, and “cell membrane” using the machine learning based Fiji Plugin *trainable WEKA segmentation*. A classifier model was trained using a minimum of eigth traces per class. The resulting probability image of “extracellular space” was auto thresholded (Otsu), converted to binary mask, selection created, and restored to the original probability image. The area was then measured as % of total area.

#### Cell stimulations for kinase profiler assay

MLS-G2 cells were seeded at 7 × 10^6^ cells per 10 mm^2^ cell culture dish in normal growth medium. After 1 h cells were transfected with negative control Gapmers or OXTR-specific Gapmers. After 24 h, the normal growth medium with the Gapmers was removed and for 2 h replaced by a serum-reduced medium (RPMI + 0.1% FCS) containing again Lipofectamine and the according Gapmers in identical concentration. The transfected cells were then stimulated with 100 nM of OXT, Atosiban, or TGOT for 10 min. Non-treated cells are indicated as vehicle (VEH). After 10 min, proteins were isolated and blotted onto a capture-antibody spotted membrane, according to the Kinase Array protocol (ARY003C, R + D Systems, Minneapolis, USA). Images were recorded by a Vilber Western Blot Imager and the membrane was analyzed using Fiji ImageJ.

#### Data acquisition and analysis

The data about cancer-related OXTR mutations were extracted from the online database cBioPortal for cancer genomics (Cerami et al., 2012; Gao et al., 2013). The term “OXTR” was queried using data from 22,882 patients/23,945 samples from a total of 31 studies as listed in Supplementary Table 1.

#### Statistics

Data were analyzed either by two-way/one-way ANOVA followed by Holm-Sidak *post-hoc* correction or Chi-square test with nominal data (sphericity assay) using GraphPad Prism 8. The variance between groups was similar, normal distribution was tested by Kolmogorov-Smirnov and Shapiro-Wilk test, and statistical significance was accepted at *p* < 0.05. N numbers represent biological replicates, technical replicates are additionally indicated. Data are represented as mean + SEM.

## Results

**Figure 1:**
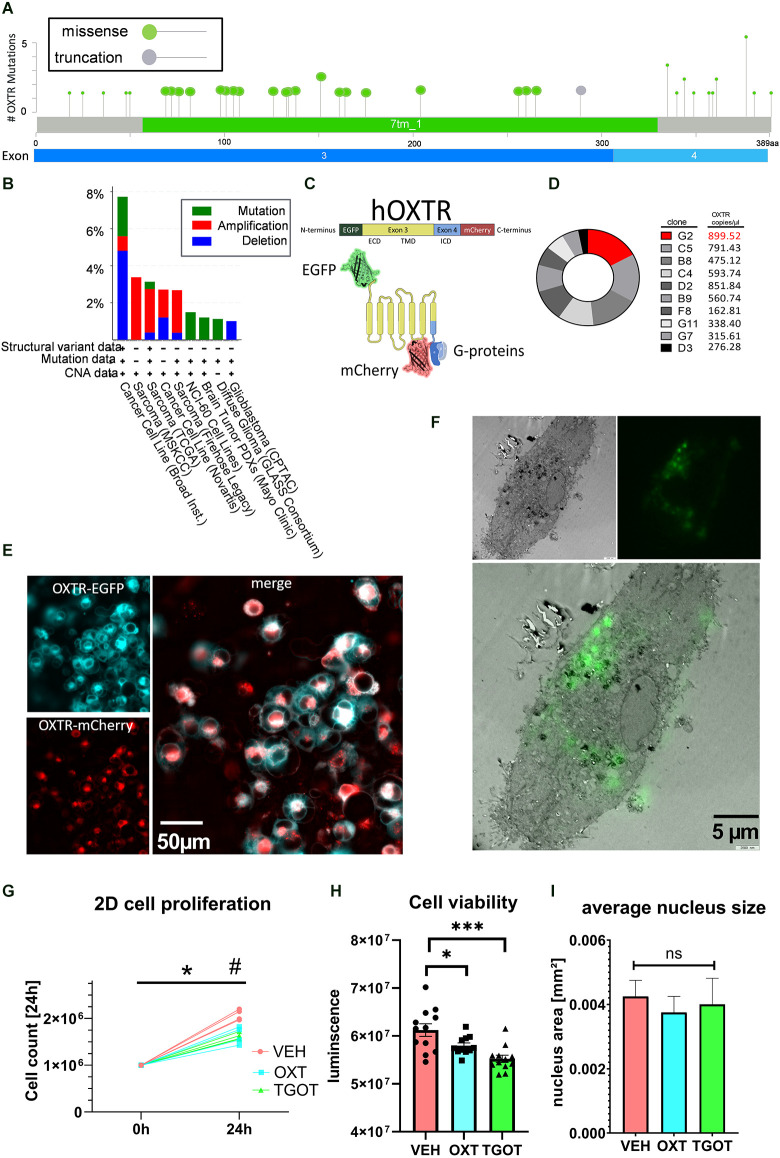
Establishment of labeled OXTR-overexpressing liposarcoma cells MLS-G2. **(A)** Localization of exonic sarcoma/carcinoma-related missense mutations or truncation within the human *OXTR* gene. Twenty missense mutations are localized in the transmembrane domain (Exon 3), and 10 missense mutations are localized in the intracellularG-protein-binding C-terminal domain. Source: cBioportal, data extracted from 23,945 samples from 31 different studies. **(B)** cBioPortal-extracted data on the occurrence of *OXTR* variants from different sources. Cancer cell lines and sarcoma tissue are represented with 8% and 3%, respectively, indicating a minor but consistent basis for the use of the liposarcoma cell line MLS-402 for *OXTR* overexpression. **(C)** Schematic representation of the labeled human *OXTR* with N-terminal EGFP and C-terminal mCherry. Exon 3 encodes the extracellular domain (ECD) and the transmembrane domain (TMB), whereas exon 4 encodes most of the intracellular domain (ICD) with the G-protein binding site. **(D)** Comparison of the expression levels of the lentiviral *EGFP-OXTR-mCherry* construct in 10 different clones of MLS-402 liposarcoma cells analyzed by dPCR as absolute numbers in copies per microliter. The clone MLS-G2 showed the highest expression of the *OXTR* construct and was therefore chosen for further experiments. **(E)** Visualization of the OXTR N-terminal EGFP (cyan) and C-terminal mCherry (red) labels. The EGFP-fluorescent signal is mainly situated in the cellular periphery, indicating a cell membrane incorporation of the OXTR; the C-terminal mCherry signal is evenly distributed in the cytoplasm, interspersed with perinuclear accumulations. **(F)** Correlative light electron microscopy (CLEM) micrograph (3,000x magnification) of an OXTR-EGFP expressing MLS-G2 cell. OXTR expression is detected perinuclear and at the cell membrane, implying functional expression. **(G)** The cellular proliferation rate of MLS-G2 cells decreases under stimulated (OXT, TGOT, both 100 nM) conditions compared to normal (control) conditions in a 2D culture after 24 h of treatment. Two-way ANOVA, time × treatment, significant interaction *F*_(2, 9)_ = 15.45, *p* = 0.0012, Tukey *post hoc* correction *p < 0.0001 vs. time point 0, ^#^*p* = 0.0001 vs. other treatment groups, *n* = 4 biological replicates. **(H)** Cell viability decreases under stimulated (OXT, TGOT, both 100 nM, 24 h) conditions. One-way ANOVA, *F*_(2, 32)_ = 10.26, *p* = 0.0004. Holm-Sidak *post hoc* correction ***P = 0.0002, *p = 0.044, *n* = 12 biological replicates. **(I)** Average nucleus size is not affected by the OXT or TGOT treatment (100 nM, 24 h) under 2D conditions. ns= not significant.

When queried for “OXTR” of approx. 24,000 samples from 31 registered studies, the cancer biology database cBioportal identified 20 exonic missense mutations and one truncation at the end of exon 3 (Figure 1A; Cerami et al., 2012; Gao et al., 2013), all of which have been associated with different types of sarcoma/carcinoma/glioma (for a complete list see Supplementary Table 1). Twenty out of 35 missense mutations lie within the large transmembrane domains of Exon 3. Interestingly, 10 out of 35 missense mutations lie within the relatively short C-terminal intracellular G-protein binding domain, and one truncation mutation occurs at the end of Exon 3, evoking the loss of Exon 4. Although not highly ranked among carcinogenic genes (Figure 1B), the OXTR protein does contribute to the regulation of proliferation and migration in a variety of different tissues (Cassoni et al., 2001), with a special emphasis on the Exon 4-encoded C-terminus.

In this study we have made use of the human liposarcoma cell line MLS-402, which we tested for endogenous *OXTR* expression and found it to be below a reliable detection level using qPCR (C_q_ > 45, data not shown). However, basal low-level expression of endogenous *OXTR* cannot be excluded. A lentiviral construct was transduced containing the human *OXTR* (based on the sequence NM_001354654.1). To detect *OXTR* expression and monitor exon 4 expression, we labeled the N-terminus of the *OXTR* sequence with EGFP and the C-terminus with mCherry (Figure 1C). Expression efficiency levels between different clones were determined by digital PCR (dPCR). Clone G2 showed the highest level of *OXTR* transcript (Figure 1D) and was, therefore, chosen for further experiments. The clonal culture will be from here on referred to as MLS-G2. Correct expression of the OXTR protein was visualized by live cell microscopy (Figure 1E), and correlative light electron microscopy (CLEM, Figure 1F), showing mostly membrane expression, with some perinuclear vesicular accumulations, representing the normal expression/recycling cycle of the OXTR (Conti et al., 2009). The general proliferation rate and morphology was identical to the mother cell line MLS-402, indicating no deleterious effects of the *OXTR* gene insertion. When stimulated under classical 2D conditions with 100 nM OXT or the specific OXTR agonist TGOT (100 nM), proliferation rates dropped significantly, indicating a correct membrane insertion of a functional receptor (Figure 1G). Moreover, cell viability determined by the ATP content decreased significantly compared to vehicle-treated cells (Figure 1H), again indicating a functional OXTR and an anti-proliferation effect of OXT in the MLS-G2 cell line. In line with an anti-proliferative effect in 2D culture, average nucleus size, a rough indicator for cell division (Webster et al., 2009) remained constant (Figure 1I).

Next, we generated spheroids from MLS-G2 cells to determine the effects of OXT and other OXTR ligands, namely Atosiban and TGOT, on basic cellular processes in a 3D environment. MLS-G2 cells begin spheroid formation already after 6–8 h and show fully formed spheroids after 24 h. Pilot experiments revealed that the spheroids are stable for 7 days and disintegrate thereafter, but already stop responding to treatment after 3 days (Supplementary Figure 1A). Based on those data, we designed our experiments accordingly to encompass 24 h of treatment where possible, or 3 days of treatment maximum. Therefore, our treatment (ligands and Gapmers) is present during the formation of spheroids, with Gapmers preceding the presence of ligands for 2 h. To control for transfection artifacts, we employed a non-specific negative control (n.c.) Gapmer and evaluated the Gapmer efficacy by dPCR with Exon 4/or Exon 2–3-specific probes, and mCherry and EGFP-specific quantitative Western blot (Supplementary Figures 1B,C).

**Figure 2:**
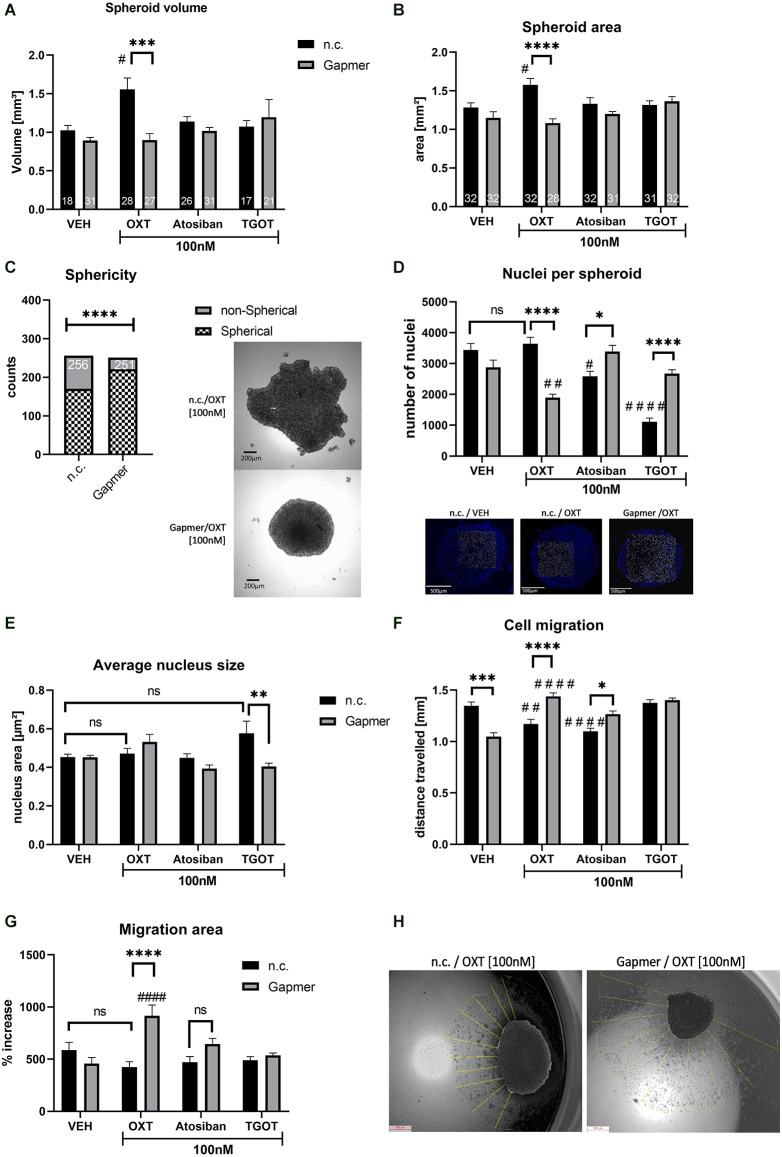
Effects of OXTR truncation and ligand treatment (OXT, Atosiban, TGOT) on cells cultured 3D in spheroids. Legend for all graphs: # = significant vs. the respective VEH control; * = significant vs. respective ligand treatment; data represented as mean + SEM; n.s. = not significant; n.c. = negative control Gapmer; Gapmer = OXTR-specific Gapmers inducing exon 4 skipping. **(A)** Spheroid volume is increased 1.5-fold by 100 nM OXT (*p* = 0.0191), an effect that is reversed by Gapmer pre-treatment (*p* = 0.0001). 100 nM Atosiban or 100 nM TGOT have no effect on spheroid volume (two-way ANOVA, Gapmer × Ligand treatment, significant interaction *F*_(3,191)_ = 4.865, *p* = 0.0028, Tukey *post hoc* correction, only spheroids with sphere-like shape are included, resulting in = 17–31, as indicated in bars). **(B)** Spheroid area corroborates the validity of the spheroid volume data. 100 nM OXT increases the spheroid surface area to ~1.6 mm^2^, Gapmer pre-treatment reverses this increase to basal levels (~1.3 mm^2^, two-way ANOVA, Gapmer × ligand treatment, significant interaction *F*_(3,242)_ = 5.933, *p* = 0.0006, Tukey *post hoc* correction, ^#^*p* = 0.031, ****p < 0.0001, *n* = 28–31, as indicated in bars). 100 nM of Atosiban and TGOT have no effect on the spheroid surface area. **(C)** Sphericity of spheroids indicates the ability to form proper cell-cell contacts and therefore round spheroids. All n.c. treated spheroids combined show a lower number of spherical spheroids than all Gapmer-treated spheroids combined, indicating an overall effect of the OXTR-intracellular domain on cell-cell contacts (Chi-square test, df = 33.64, 1; *p* < 0.0001, *z* = 5.8, two-sided Fisher’s exact test *p* < 0.0001; *n* = 251–256, as indicated in bars). **(D)** The number of nuclei in spheroids indicates cell count and therefore proliferation rate. 100 nM n.c./OXT-treated spheroids do not show an increased nucleus count per spheroid, but a significant decrease in Gapmer/OXT-treated spheroids compared to Gapmer/VEH (*p* = 0.0057) and n.c./OXT-treated spheroids (*p* < 0.0001). 100 nM Atosiban decreases cell count significantly (*p* = 0.0257), and Gapmer treatment reversed this effect back to basal levels (*p* = 0.043). 100 nM TGOT exerted the strongest effect on cell count by reducing nuclei per spheroid to one-third of the VEH levels (*p* < 0.0001), but Gapmer treatment reversed this effect to basal levels (*p* < 0.0001 vs. n.c./TGOT). Two-way ANOVA, Gapmer × ligand treatment, significant interaction *F*_(3,72)_ = 33.57, *p* < 0.0001, Tukey *post hoc* correction, *n* = 10. **(E)** Average nucleus size indicates cell proliferation or cell death. No effect of the 100 nM OXT, Atosiban, or TGOT treatment was detectable, however, Gapmer pre-treatment significantly decreased average nucleus size of TGOT treated cells (*p* = 0.0044). Two-way ANOVA, Gapmer × ligand, significant interaction *F*_(3,72)_ = 5.127, *p* = 0.0029, *n* = 10. **(F)** Cell migration is inhibited by OXTR activation. 100 nM of OXT (*p* = 0.0067, black bar) and Atosiban (*p* < 0.0001, black bar) reduce the distance traveled by ~300 μm. Gapmer pre-treatment under VEH conditions reduces cell migration significantly (*p* < 0.0001, gray bar), but increases the distance traveled compared to its respective ligand treatment (OXT *p* < 0.0001; Atosiban *p* = 0.01) and to its respective VEH control (OXT *p* = 0.0067; Atosiban *p* < 0.0001). Two-way ANOVA, Gapmer × ligand treatment, significant Interaction *F*_(3,632)_ = 26.93; *p* < 0.0001, Tukey *post hoc* correction, *n* = 8 biological replicates, 10 technical replicates per biological replicate. **(G)** Migration area is assessed by measuring the area of the outgrown cell layer, which corroborates the cell migration data. Gapmer pre-treatment increases the cells’ ability to migrate, resulting in a larger area covered in Gapmer/OXT-treated spheroids compared to the n.c./OXT group (*p* < 0.0001), or to the n.c./VEH group (*p* < 0.0001). Two-way ANOVA, Gapmer × ligand treatment, significant interaction *F*_(3,56)_ = 9.298, *p* < 0.0001, Tukey *post hoc* correction, *n* = 8. **(H)** Visualization of the migrated cell layer originating from the mother spheroid. Cell migration was measured from the overlayed day 0 spheroid border to the border of the cell layer, as indicated by yellow lines.

When transfected with n.c. Gapmers and treated with 100 nM OXT for 24 h, spheroid volume and area increased significantly at 56% and 23%, respectively, compared to n.c./vehicle-treated spheroids (black bars, Figures 2A,B). In case spheroids deviate from a sphere-like shape, volume calculation becomes unreliable. However, the spheroid surface area can be accurately measured independent of the spheroid shape and represents a sound method to assess spheroid size. Sphericity, which would allow volume calculation in all spheroids, was not evenly distributed among the groups. When compared between n.c. and Gapmer treated spheroids, regardless of the ligand treatment, the sphericity of spheroids was increased in Gapmer-treated cells (Figure 2C). Consequently, vehicle (VEH, H_2_O) treatment causes cells to form dense clusters with a macroscopic spherical shape but blocking the OXTR with Gapmers induces a less dense cell cluster displaying an irregular macroscopic shape.

Furthermore, the number of nuclei per spheroid, indicative of cell count, was not increased by the 100 nM OXT treatment (Figure 2D). In addition, nucleus size, indicative of increased cell size to account for increased spheroid size, was not increased by 100 nM OXT (Figure 2E). If spheroid size increases, but neither cell number nor cell size increases, the only possible explanation is a decreased cell density within the spheroid. Indicative of altered cell-matrix attachment, when placed on an adherent surface, OXT-treated cells migrated less far from their spheroid than VEH-treated cells. Decreased cellular migration was determined as the average migration distance the cells traveled from their spheroid (Figure 2F) and as the area of the cell layer surrounding the spheroid (Figures 2G,H).

Interestingly, the effects of OXT on spheroid volume and area were blocked by the OXTR specific Gapmers (gray bars, Figures 2A,B) that induced exon 4 skipping and loss of the intracellular G-protein binding domain of the OXTR. Consequently, the cells’ ability to attach to the extracellular matrix was restored, resulting in increased migration distance and area of Gapmer/OXT-treated cells, compared to Gapmer/VEH-treated cells (Figures 2F,G).

Atosiban acts as a biased OXTR agonist that selectively activates the Gα_i_-protein but antagonizes the Gα_q_ OXTR and V1a receptors. In our cell model, n.c./Atosiban [100 nM] treatment does neither alter the spheroid size, nor nucleus size, however, the number of nuclei in a spheroid is reduced, indicating a reduced proliferation rate (Figure 2D). Cellular migration, as measured by distance traveled was also significantly reduced (Figures 2D,F). Taken together, the actions of Atosiban *via* the Gα_i_-protein pathway reduce proliferation in a spheroid and inhibit cellular migration.

When pre-treated with the OXTR-specific Gapmers, Atosiban can no longer activate the Gα_i_-pathway and is unable to reduce proliferation (Figure 2D, gray bars) or cell migration (Figure 2F, gray bars), highlighting the importance of the intracellular G-protein binding domain for this effect. The absence of an effect on spheroid size indicates that the cell-cell attachment, as altered by OXT, depends on an alternative G-protein pathway, e.g., Gα_q_ or Gα_o_.

In contrast to OXT but in accordance with Atosiban, TGOT did not alter spheroid volume or area (Figures 2A,B black bars). This difference indicates slight binding differences between OXTR agonists, as discussed below in the discussion section.

However, 100 nM TGOT was most effective in reducing nuclei count per spheroid to an about three-fold reduction (Figure 2D, black bar), with a non-significant trend towards increased nucleus size (Figure 2E, black bar). Gapmer pre-treatment reversed both effects, i.e., increased the nuclei count to basal levels (Figure 2D, gray bar) and significantly decreased the average nucleus size, compared to n.c./TGOT treated spheroids, back to basal levels (Figure 2E, gray bar).

**Figure 3:**
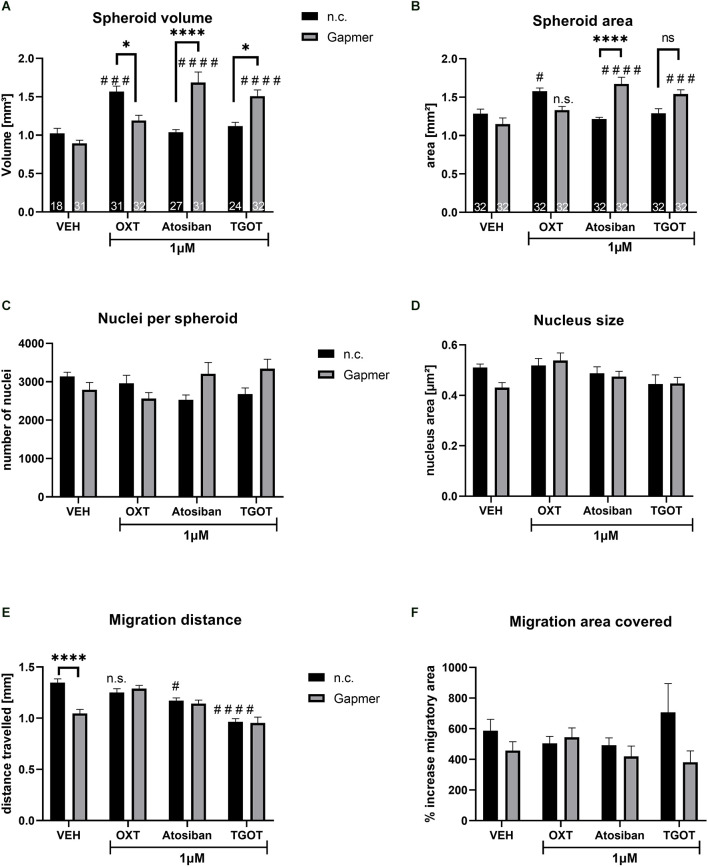
Spheroid size, nucleus count and size, and cellular migration caused by 1 μM of ligand treatment (OXT, Atosiban, TGOT) under negative control (n.c.) or OXTR Gapmer (Gapmer) conditions. Legend for all graphs: # = significant vs. the respective VEH control; * = significant vs. respective ligand treatment; n.s. = not significant; data represented as mean + SEM; n.c. = negative control Gapmer; Gapmer = OXTR-specific Gapmers inducing exon 4 skipping. **(A)** Spheroid volume was not affected by Gapmer pre-treatment under VEH conditions. When stimulated by n.c./OXT, spheroid volume increased significantly compared to n.c./VEH (*p* = 0.0005), and Gapmer pre-treatment reversed the spheroid volume back to basal levels (*p* = 0.0108). n.c./Atosiban treatment did not alter spheroid volume, but Gapmer pre-treatment increased it 1.68-fold (*p* < 0.0001) compared to n.c./Atosiban and Gapmer/VEH. The treatment of n.c./TGOT did not affect spheroid volume, but pre-treatment with Gapmers significantly increased it compared to Gapmer/VEH (*p* < 0.0001) and n.c./TGOT (*p* = 0.0161). Two-way ANOVA, Gapmer × ligand treatment, significant interaction *F*_(3,218)_ = 18.10, *p* < 0.0001; *n* = 18–27 as indicated in bars. **(B)** Spheroid area data parallels spheroid volume, with no effect of Gapmer pre-treatment under VEH conditions, but increased spheroid area by n.c./OXT stimulation (*p* = 0.0150) compared to n.c./VEH. Gapmer pre-treatment reversed the effect so that the spheroid area is no longer significantly different from the Gapmer/VEH. However, unlike spheroid volume, the effect on the area is too small to be significantly different from the n.c./OXT group. Neither n.c./Atosiban nor n.c./TGOT altered spheroid area, but Gapmer pre-treatment increased it significantly (*p* < 0.0001 and *p* = 0.0002). Two-way ANOVA, Gapmer × ligand treatment, significant interaction *F*_(3, 248)_ = 14.94, *p* < 0.0001; *n* = 32. **(C)** The number of nuclei per spheroid was not affected by 1 μM treatment of OXT, Atosiban, or TGOT. **(D)** The size of nuclei within spheroids was not affected by 1 μM of OXT, Atosiban, or TGOT. **(E)** Migration distance was decreased by the Gapmer treatment (*p* < 0.0001) only in the VEH group, but in none of the other treatment groups. Atosiban (*p* = 0.0217) and TGOT (*p* < 0.0001) reduced the migration distance significantly and Gapmer pre-treatment had no effect on this reduction. **(F)** A significant overall effect of Gapmer treatment on migration area was detected in the two-way ANOVA, however, none of the comparisons after Tukey *post hoc* correction were significant. Two-way ANOVA, ligand × Gapmer treatment, Gapmer treatment *F*_(1,55)_ = 4.330; *p* = 0.0421, *n* = 8.

In contrast to OXT or Atosiban treatment, the concentration of 100 nM TGOT was not effective to alter cellular migration (Figures 2F,G). As shown in the following Figure 3, the higher TGOT concentration of 1 μM decreased cellular migration, indicating that TGOT is indeed effective, but only at higher concentrations.

Similar to the 100 nM treatment regimen (see Figure 2A), treating spheroids with 1 μM OXT increased spheroid volume, but 1 μM Atosiban and TGOT had no effect (Figure 3A). The OXT-induced volume increase was blocked by Gapmer pre-treatment and reversed the volume back to basal levels. However, pre-treatment with Gapmers increased the volume in Atosiban and TGOT treated spheroids. This indicates: (1) that the OXT-induced effect is mediated by the intracellular G-protein domain of the OXTR; and (2) that 1 μM and 100 nM OXT activate a unique pathway that is disregarded by the other ligands in both concentrations. Spheroid area measurements corroborated the spheroid volume data (Figure 3B), granting the validity of the assessment method. Cellular proliferation within the spheroid was not affected by the 1 μM treatment with OXT, Atosiban, or TGOT, as indicated by the number (Figure 3C) and size (Figure 3D) of spheroidal nuclei. Migration from the spheroid onto a 2D surface was reduced by the Gapmer pre-treatment under VEH conditions, and by 1 μM Atosiban and TGOT stimulations (Figure 3E). However, Gapmer pre-treatment had no effect on the Atosiban and TGOT-induced reduction. This seemingly contradictory result must be taken with caution, as the slightly different assessment method of “migration area covered” (Figure 3F) revealed only an overall significant effect of the Gapmer treatment but did not detect any significant difference between the groups. This inconsistency suggests that the mathematical significant effects of the 1 μM OXT, Atosiban, and TGOT treatment are biologically questionable.

**Figure 4:**
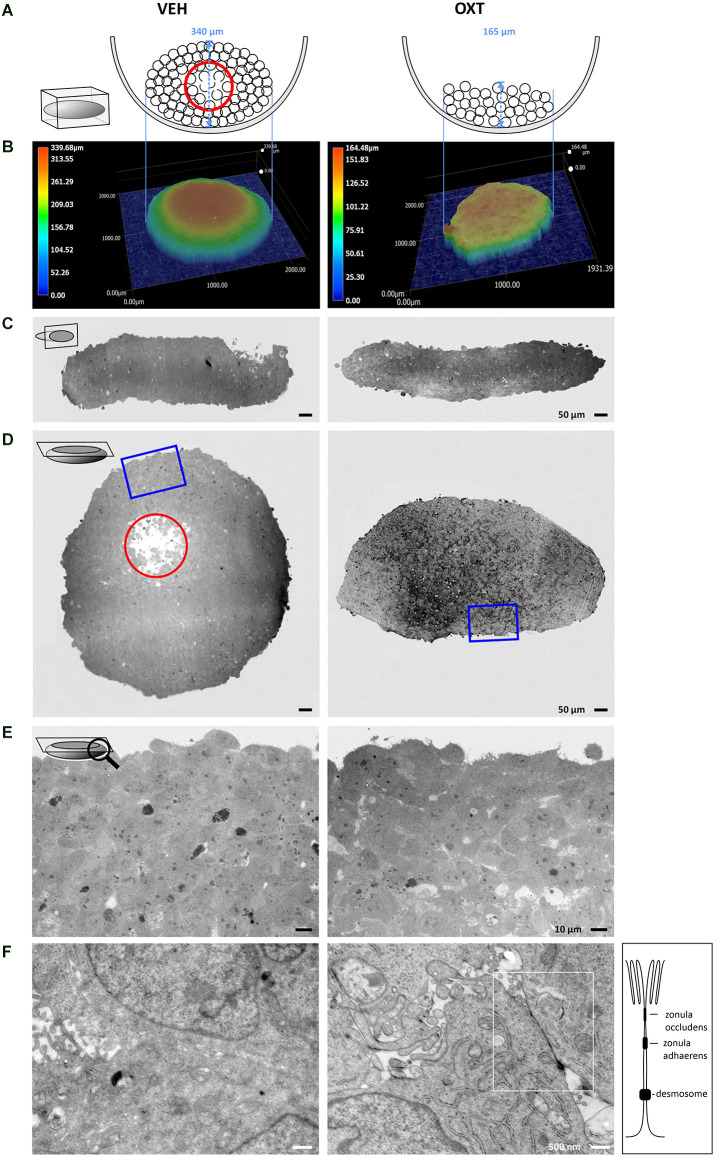
Images of VEH-treated (left column) vs. OXT-treated (right column, 100 nM for 24 h) spheroids shown in different orientations and magnification using TEM and digital 3D-microscopy. **(A)** Schematic representation of the OXT-induced effect on spheroid formation. The U-shaped well bottom is shown in the side-view. Black circles represent individual cells, a red circle indicates the apoptotic zone within the round spheroid. Please note the absence of an apoptotic zone in the flat OXT-treated spheroid. **(B)** Representative 3D images of VEH vs. OXT-treated spheroids. VEH-treated spheroids resemble a gravity-condensed ball-shape, whereas OXT-treated spheroids are irregular in shape and less densely packed. **(C)** Diagonal 250× TEM micrographs of 70 nm sections of VEH or OXT-treated spheroids. VEH-treated spheroids appear more uniform, indicating a reduced intercellular space, whereas OXT-treated spheroids show clear intercellular spacing. **(D)** Longitudinal 250× micrographs of 70 nm sections of the identical spheroids used in **(C)**. The red circle indicates the apoptotic zone, which is only apparent in the VEH-treated spheroid; the blue square indicates the magnified zone in **(E)**. **(E)** 400x magnified micrographs of the spheroid shown in **(D)**. VEH-treated spheroids show a high cell density with sparse intercellular spacing, whereas OXT-treated spheroids show a less densely packed cell clustering. **(F)** 12,000x magnification reveals atypical and barely distinguishable cell-cell contacts in VEH-treated spheroids, but very regular and evenly-distributed classical tight junctions in OXT-treated spheroids. Additional TEM micrographs showing more OXT-induced tight junctions or the lack thereof in VEH-treated spheroids can be provided upon request.

A top-down view of spheroids is insufficient to correctly interpret the true 3-dimensional cellular organization of the spheroid, therefore we employed different methods to visualize the shape and consistency of the spheroid. As shown in the schematic illustration of Figure 4A, the growth of the spheroids is dependent on the treatment with OXT. These differences can be detected with digital 3D microscopy, which revealed the true surface shape of the spheroids by Z-stack stitching of 200 × magnification images and digital reconstruction in three dimensions. This approach also allows to visualizing ripples and wrinkles on the spheroid surface that would be invisible in a top-down view. As expected, VEH-treated spheroids show a smooth, “ball-like” shape, whereas 100 nM OXT-treated spheroids reveal a rough and uneven surface. As an example of the importance of 3D-imaging, the representative right image in Figure 4B of the 100 nM OXT-treated spheroid shows a small hump on the left side, which would have been missed with the other techniques.

For further investigations, the spheroids were cut in ultrathin sections from two perpendicular planes (diagonal and longitudinal) to evaluate them by transmission electron microscopy (TEM). These orientations of the electron micrographs reveal the overall shape of the spheroids, as well as the cell density below the spheroid surface. The untreated (VEH) spheroids show a round, “ball-like” shape, a high cell density, and very minute extracellular space, except for the apoptotic zone at the near-center of the spheroid (red circle, Figures 4C,D). The nutrient and oxygen supply decreases towards the inner zones of the spheroid, creating a survival gradient from the proliferating outer zone towards the inner-most apoptotic center (Ryu et al., 2019). In contrast, OXT-treated spheroids show a decreased cellular density and evenly distributed extracellular spaces (see Supplementary Figure 2 for quantification of the extracellular space and Supplementary Figure 3 for the spatial distribution of tight junctions within a spheroid). The macroscopic shape of the OXT-treated spheroid deviates from the “ball-like” shape of the VEH-treated spheroids, thereby increasing its surface. In addition, higher magnification reveals the widespread OXT-induced formation of tight junctions and protrusions of the “luminal” side of the cells that reach into an enlarged extracellular space. This indicates well-organized cell-cell contacts, which level out the nutrient and oxygen gradient even further, resulting in an absent apoptotic zone (Figures 4E,F).

**Figure 5:**
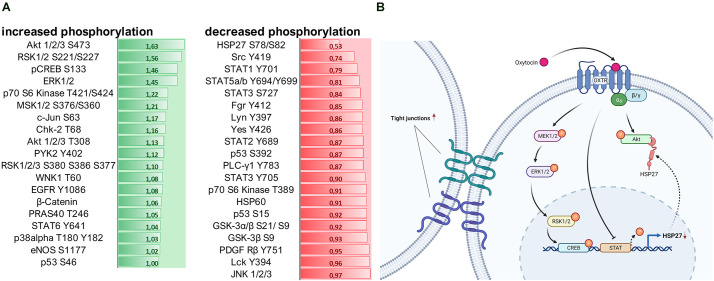
**(A)** Assessment of 100 nM OXT-induced signaling cascade activation by quantification of kinase phosphorylation. Data is shown in fold change (green = increase; red = decrease) compared to basal VEH-treated cells. A fold change of greater than ± 0.2 is considered relevant. Signaling cascades involved in cell survival are upregulated, and cascades involved in cell-cell adhesion and cell-matrix interaction are downregulated. **(B)** Schematic overview of the intracellular signaling cascades activated by the OXTR and their effect on tight junction formation. Created with www.BioRender.com.

Lastly, by using a kinase phosphorylation profiler assay we evaluated whether OXT influences the regulation of signaling cascades associated with cell survival and cell-cell adhesion. Most notably, OXT increased the phosphorylation of Akt 1/2/3 S47 by 63%, whilst it dephosphorylated heat shock protein 27 (HSP27) by 47% (Figure 5A). For a better understanding, we summarized the OXT-induced intracellular pathways and their outcomes on cell-cell interaction in a schematic overview (Figure 5B).

## Discussion

The neuropeptide OXT has been implicated in a multitude of behavioral and physiological effects, and our understanding of the intracellular signaling cascades that are responsible for those effects is ever-growing (Grinevich and Neumann, 2021). However, the extent to which OXT affects intracellular processes is yet underappreciated, as evidenced by our and other groups’ recent discoveries (Zatkova et al., 2019; Meyer et al., 2020, 2022; Reichova et al., 2020; Salehi et al., 2021). For instance, OXT modulates cellular morphology, which is one important aspect of intercellular communication, especially in neurons and astrocytes (Meyer et al., 2018; Falougy et al., 2019). Cellular ATP content and mitochondrial respiration rate, which support morphological rearrangements, are further enhanced by OXTR-induced signaling cascades (Meyer et al., 2020). Those neuron-specific OXT effects are translatable to other cell types, especially cancer cells, where OXT affects proliferation and cellular migration in a cell-type-specific way (Cassoni et al., 2004; Cattaneo et al., 2008). As mentioned in the introduction, it is nearly impossible to predict the effect OXT has on a specific cell type. However, what makes up the difference between cell types that causes OXT to act in sometimes diametrical opposing ways is yet unknown. One aspect to solve this issue could be the removal of culturing artifacts. While most of the early studies on OXT and cancer have been conducted in classical two-dimensional cultures (Cassoni et al., 2004), we know nowadays that a 3D environment and cell-matrix interactions alter basic cellular characteristics and can affect the cellular response to certain stimuli (Jensen and Teng, 2020; Pandamooz et al., 2020). Consequently, we chose in our study a cellular model consisting of a cancer type that shows OXTR dysregulation, i.e., liposarcoma cells, and a 3D spheroid culturing method, which helps to reduce and replace *in vivo* animal experiments, and allows to monitor cell-cell interaction (tight junction formation) in a natural environment. The cellular assays we ran with our liposarcoma spheroids, e.g., proliferation or migration, are not only meaningful for metastases formation in cancer biology, but also for neuronal migration and survival in the brain, wound healing, etc. In that sense, we hope that this study prompts further research into OXT-induced effects in a 3-dimensional culture setting with other cell types.

Since Gerald Gimpls’ and Falk Fahrenholzs’ review about the oxytocin receptor in 2001 (Gimpl and Fahrenholz, 2001), according to PubMed 240 articles have been published that address “oxytocin receptor signaling” and the binding of different types of G-proteins upon exposure to different ligand concentrations or biased agonists (Busnelli and Chini, 2018; Jurek and Neumann, 2018). Those different types of G-proteins, namely Gα_i_, Gα_q_, and Gα_o_, activate different sets of signaling cascades, including the transactivation of other receptors (EGFR; Blume et al., 2008) or channels (TrpV1; van den Burg et al., 2015). The most commonly studied OXTR-coupled pathway is the MAPK pathway, consisting of ras, raf, MEK1/2, and ERK1/2 (Blume et al., 2008; Jurek et al., 2012, 2015), acting on downstream transcription factors like CREB (Tomizawa et al., 2003; Jurek et al., 2015) and MEF2A (Meyer et al., 2018, 2020; Winter et al., 2021). Here, in this study, we aimed to broaden the scope of OXTR-coupled pathways by using a kinase array, which revealed a strong activation of the Akt (also known as protein kinase B) pathway. Akt is known to phosphorylate the transcription factor CREB, which drives the expression of survival genes (Walton and Dragunow, 2000), but is also involved in proliferation, metabolism, and angiogenesis (Hoxhaj and Manning, 2020). Closely related to the Akt pathway is the heat shock protein 27 (HSP27), which directly orchestrates the formation of filamentous actin (F-actin). F-actin is a prerequisite for coupling to the β-integrin complex that connects the cell to the extracellular matrix (ECM; Gerthoffer and Gunst, 2001). We have previously shown that OXT treatment reduces β-integrin protein expression (Meyer et al., 2020) and that it reduces the formation of F-actin fibers (unpublished personal communication with Prof. Dr. Sareh Pandamooz). Consequently, we can now provide the full pathway from the activated OXTR, to reduced HSP27 phosphorylation, reduced F-actin formation, and reduced β-integrin expression, ultimately causing diminished cellular migration.

In addition, we are able to add another novel aspect to the OXTR-coupled cascades, which are the STAT1–5 transcription factors. The observed OXT-induced downregulation of STAT proteins likely results in a downregulation of HSP27 protein, since HSP27 expression depends on active STAT transcription factors. This adds another layer of regulatory control of OXT-induced signaling cascades over cellular effects like F-actin formation, β-integrin function, and finally cellular migration.

Another aspect of intercellular effects was the OXT-induced formation of tight junctions between cells of the treated spheroid, unlike in VEH-treated spheroids, where densely packed cell clusters seem to only be brought to close proximity by gravity. We also describe for the first time the clear formation of tight junctions in OXT-treated spheroids, indicating a polarization of the cells towards a defined extracellular space, with a “luminal” and a “basal” side of the cells, a process described as mesenchymal to epithelial transition (MET; Jayachandran et al., 2021). This OXT-induced MET counteracts the opposing epithelial to mesenchymal transition (EMT), which generally occurs during the regular (untreated) formation of spheroids (Jeon et al., 2017). Despite the irregular shape of the OXT-treated spheroids on a macroscopic level, 12,000× magnification using TEM reveals regular and functional tight junctions between cells of the spheroid, and no apoptotic zone in the center of the spheroid. It is tempting to speculate that the formation of highly functional tight junctions and a surrounding enlarged extracellular space (“lumen”) and consequently a polarized cell morphology accelerates the nutrient transport and gas exchange within the spheroid, preventing the formation of an apoptotic zone at the inner-most core of the spheroid. This process is of special interest for the treatment of degenerative diseases where cells lose polarity, cell-cell contacts, and undergo apoptosis, e.g., as it is the case in the breakdown of the blood-brain barrier (Knox et al., 2022). Another pathological aspect that is affected by cell-cell contacts and cell polarity is metastatic tumor formation, where single cells undergo EMT, detach from the primary tumor, and invade other organs or tissues *via* the bloodstream (Ben Amar et al., 2022). As OXT opposes the EMT by inducing the formation of tight junctions and also inhibits cellular migration, it might be helpful to consider its use as an adjuvant treatment.

Having identified tight junction formation in OXT-treated spheroids by TEM micrographs, this study only contains a limited methodological approach to further analyze tight junctions, a limitation that we will address in subsequent studies.

Despite the clear effect of OXT on our spheroid model, OXT is a promiscuous molecule which can also bind other receptors (Chini et al., 2008; Nersesyan et al., 2017), therefore it is of utmost importance to control for OXTR activation with a specific and highly effective inhibitor. Pharmacological inhibitors are notorious for being unspecific for their intended target (Manning et al., 2008), and a mere knockdown of the OXTR increases the likelihood of OXT or even TGOT binding to vasopressin receptors (Sala et al., 2013) or TrpV1 channels (Nersesyan et al., 2017). In addition, the binding of different OXTR agonists can produce varying outcomes: for instance, in contrast to OXT but in accordance with Atosiban, TGOT did not alter spheroid volume or area (Figures 2A,B black bars). As TGOT has a slightly lower receptor affinity than OXT (Ki: 0.79 nM OXT vs. 6.62 nM TGOT; Chini and Manning, 2007), a higher than 100 nM TGOT concentration is required to prompt the same G-protein pathways as OXT. As previously published (Busnelli and Chini, 2018), 90–100 nM OXT is more likely to activate the Gαo-pathway, whereas concentrations between 30 and 90 nM OXT activate the Gα_i_-pathways. As TGOT has a lower affinity for the OXTR (Busnelli et al., 2013), 100 nM TGOT is more likely to activate the Gα_i_-pathway. This pathway, as observed with Atosiban, does not affect cell-cell attachments, and therefore spheroid size.

To overcome those issues, we designed a novel approach to inhibit the OXTR signaling by transiently removing the intracellular G-protein-coupling domain from the receptor *via* Gapmer-induced exon skipping, which leaves the transmembrane and ligand binding domain intact. In that way, we are able to specifically block OXTR-signaling, without increasing the likelihood of the supplemented OXT binding to other receptors. Moreover, this approach is highly selective for the OXTR against the vasopressin receptors, as it relies on Gapmer binding in the *OXTR*-specific sequence of exon 4, unlike pharmacological inhibitors which face the difficult-to distinguish similarities between OXTR and vasopressin receptors on the protein level. We have previously used Gapmers to induce alternative splicing and subsequent exon-skipping to alter the characteristics of another GPCR, the corticotropin releasing factor receptor 2α (CRFR2α) as well asthe TGF-ß receptor and confirmed their applicability *in vivo* (Peters et al., 2021; Winter et al., 2021). Therefore, we hope to prompt further use of this highly efficient and specific OXTR inhibitor for basic and applied research *in vitro* as well as *in vivo*.

In summary, this study reveals some novel aspects of OXTR-coupled intracellular signaling (STAT1-5, HSP27, Akt) that determine the ability of cells to form tight junctions within 3-dimensional spheroids and to migrate on a 2-dimensional surface. We have narrowed down the effects of 100 nM and 1 μM OXT to the OXTR, as Gapmer-induced exon skipping efficiently diminished or even reversed the effects of OXT. We also advocate for the use of digital PCR (dPCR) to reliably quantify small amounts of *OXTR* transcript, and the use of CLEM to visualize the OXTR protein on a subcellular level. Taken together, our data lays the foundation for future OXT-related studies in the field of (neuro-) degenerative diseases or tumor formation employing 3D-culturing methods like spheroids or organoids.

## Data Availability Statement

The original contributions presented in the study are included in the article/Supplementary material, further inquiries can be directed to the corresponding author.

## Author Contributions

BJ, NS, and LD contributed to the conception and design of the study. BJ wrote the first draft of the manuscript. LD wrote sections of the manuscript. All authors contributed to the article and approved the submitted version.

## Funding

Funding was provided by the Angela Schötz Keilholz foundation (grant number 3/2021).

## Supplementary Material

Supplementary Table 1 List of studies queried for “OXTR” and associated sample number per study.

Supplementary Figure 1 **(A)** Lack of effect of different doses of OXT or TGOT on spheroid volume after 3 days of culturing. N numbers are indicated in bars, ligand concentration is indicated in x-axis. Data represented as mean + SEM. **(B)** dPCR-determined absolute copy numbers of OXTR transcript downregulated 10, 2, or 0.2 nM OXTR Exon 4-specific Gapmers by compared to negative control Gapmer (n.c.). Data represented as mean + SD. **(C)** Western Blot showing EGFP and mCherry expression in Gapmer or negative control (n.c.) treated spheroids. No difference in EGFP expression is detectable, while Gapmer treatment induced a 35% downregulation of mCherry compared to n.c.

Supplementary Figure 2 Evaluation of the area occupied between the cell membranes of neighboring cells (=extracellular space in a spheroid). A machine learning image segmenting approach revealed significantly increased extracellular space in OXT-treated spheroids. Normal distribution tested by Kolmogorov-Smirnov and Shapiro-Wilk test, two-tailed, unpaired t-test *p* < 0.0001, *t* = 5,365, *n*(control) = 15, *n*(OXT) = 35.

Supplementary Figure 3 Evaluation of spatial distribution of tight junction formation. The OXT-induced increase in extracellular space and tight junction formation is independent of the position within the spheroid. Tight junctions (OXT) or cell-cell borders (controls) are similar at the center, near center, or periphery of the spheroid.
