# Supplementary material for: Oxytocin accelerates tight junction formation and impairs cellular migration in 3D spheroids: evidence from Gapmer-induced exon skipping
Source: Front Cell Neurosci. 2022 Oct 3;16:1000538. doi: 10.3389/fncel.2022.1000538 (PMC9574052; doi:10.3389/fncel.2022.1000538)
Supplement: Supplementary Table 1 — List of studies queried for “OXTR” and associated sample number per study. [file Table_1.pdf]

Study	Sample number
Pediatric Brain Cancer (CPTAC/CHOP, Cell 2020)	218
Brain Lower Grade Glioma (TCGA, Firehose Legacy)	530
Brain Lower Grade Glioma (TCGA, PanCancer Atlas)	514
Diffuse Glioma (GLASS Consortium, Nature 2019)	444
Glioma (MSK, Nature 2019)	91
Glioma (MSKCC, Clin Cancer Res 2019)	1.004
Low-Grade Gliomas (UCSF, Science 2014)	61
Merged Cohort of LGG and GBM (TCGA, Cell 2016)	1.122
Brain Tumor PDXs (Mayo Clinic, 2019)	97
Glioblastoma (CPTAC, Cell 2021)	99
Glioblastoma (Columbia, Nat Med. 2019)	42
Glioblastoma (TCGA, Cell 2013)	577
Glioblastoma (TCGA, Nature 2008)	206
Glioblastoma Multiforme (TCGA, Firehose Legacy)	619
Glioblastoma Multiforme (TCGA, PanCancer Atlas)	592
Anaplastic Oligodendroglioma and Anaplastic Oligoastrocytoma	22
Medulloblastoma (Broad, Nature 2012)	192
Medulloblastoma (DKFZ, Nature 2017)	491
Medulloblastoma (ICGC, Nature 2012)	125
Medulloblastoma (PCGP, Nature 2012)	37
Medulloblastoma (Sickkids, Nature 2016)	46
Pilocytic Astrocytoma (ICGC, Nature Genetics 2013)	96
Pheochromocytoma and Paraganglioma (TCGA, Firehose Legacy)	184
Cancer Cell Line Encyclopedia (Broad, 2019)	1.739
Cancer Cell Line Encyclopedia (Novartis/Broad, Nature 2012)	1.020
MSK-IMPACT Clinical Sequencing Cohort (MSKCC, Nat Med 2017)	10.945
NCI-60 Cell Lines (NCI, Cancer Res 2012)	67
Sarcoma (MSK, 2022)	2.138
Sarcoma (MSKCC/Broad, Nat Genet 2010)	207
Sarcoma (TCGA, Firehose Legacy)	265
Sarcoma (TCGA, PanCancer Atlas)	255
