# Supplementary figures and images for: Oxytocin accelerates tight junction formation and impairs cellular migration in 3D spheroids: evidence from Gapmer-induced exon skipping
Source: Front Cell Neurosci. 2022 Oct 3;16:1000538. doi: 10.3389/fncel.2022.1000538 (PMC9574052; doi:10.3389/fncel.2022.1000538)

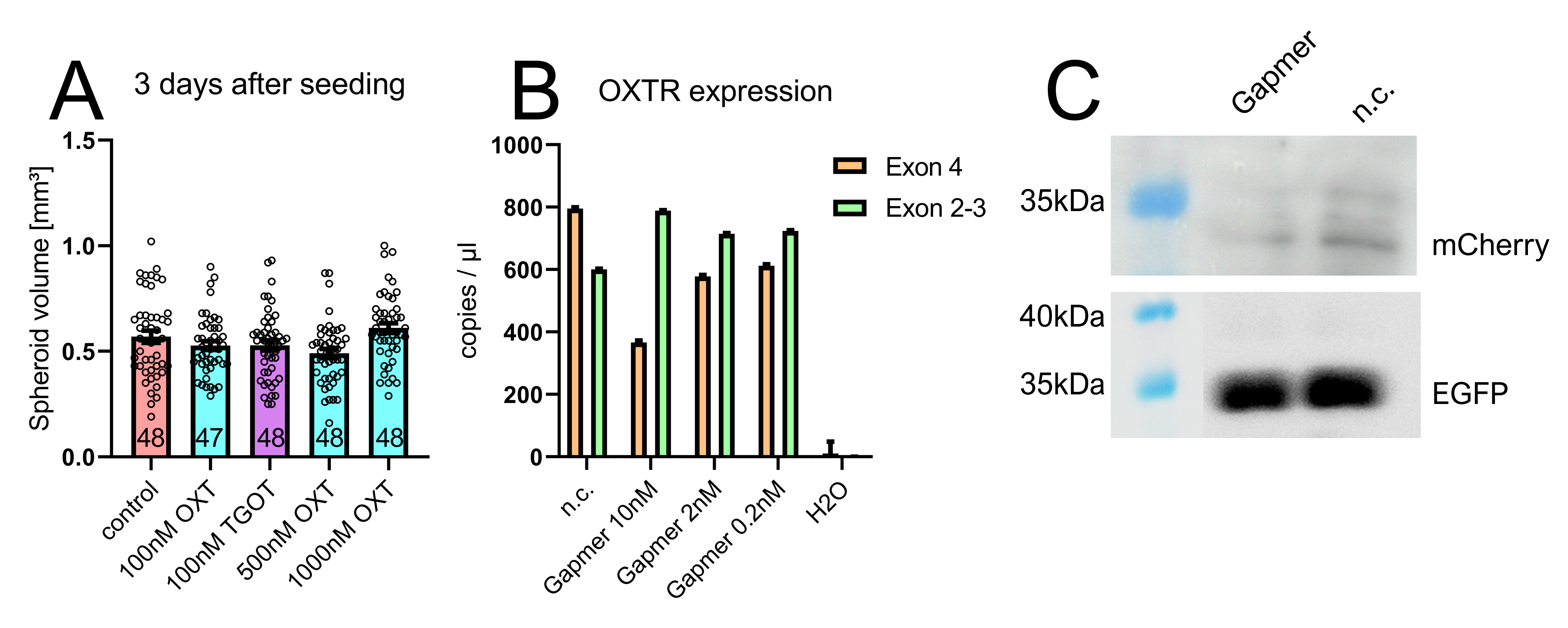

Supplement: Supplementary Figure 1 — (A) Lack of effect of different doses of OXT or TGOT on spheroid volume after 3 days of culturing. N numbers are indicated in bars, ligand concentration is indicated in x-axis. Data represented as mean + SEM. (B) dPCR-determined absolute copy numbers of OXTR transcript downregulated 10, 2, or 0.2 nM OXTR Exon 4-specific Gapmers by compared to negative control Gapmer (n.c.). Data represented as mean + SD. (C) Western Blot showing EGFP and mCherry expression in Gapmer or negative control (n.c.) treated spheroids. No difference in EGFP expression is detectable, while Gapmer treatment induced a 35% downregulation of mCherry compared to n.c. [file Image_1.tif]

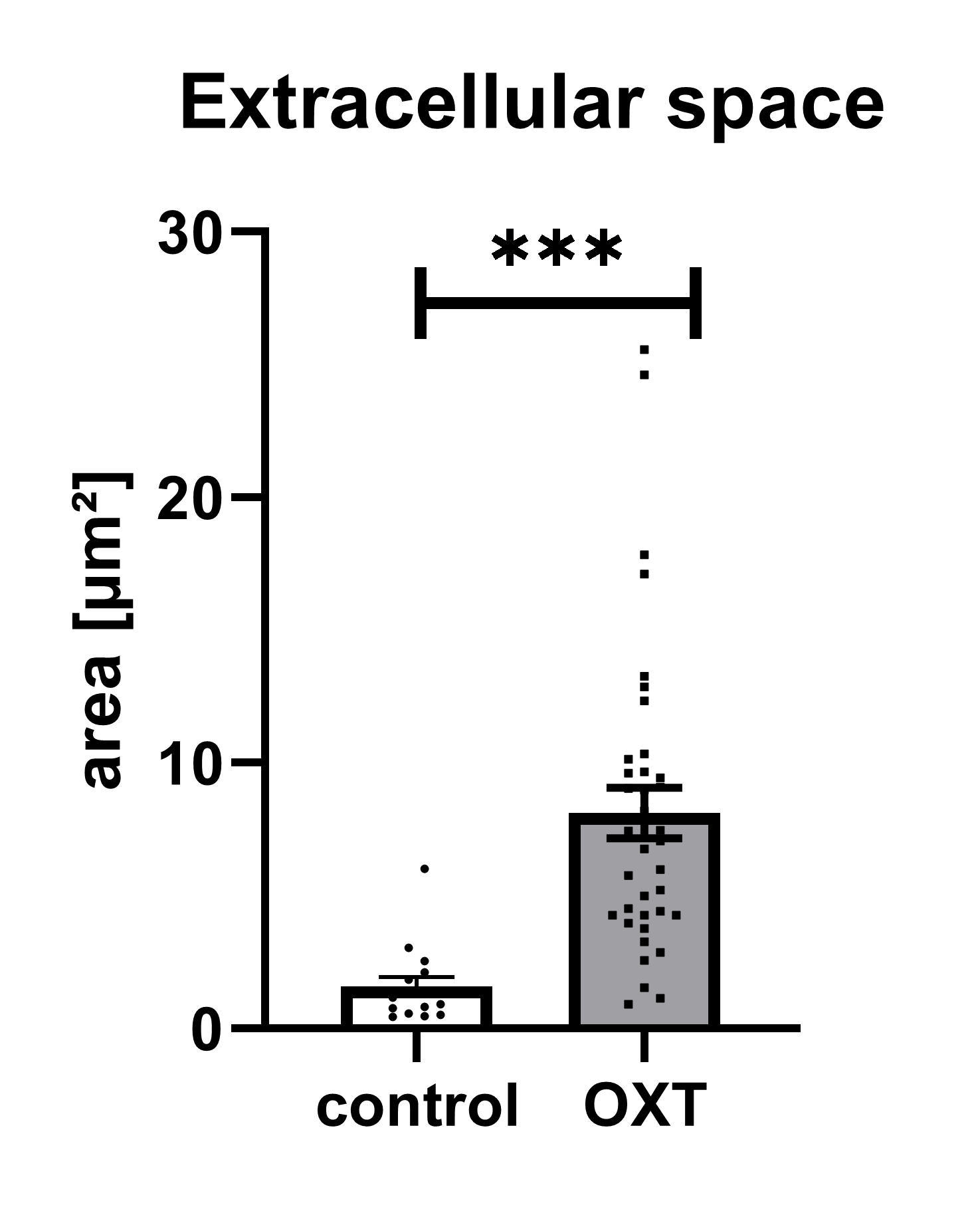

Supplement: Supplementary Figure 2 — Evaluation of the area occupied between the cell membranes of neighboring cells (=extracellular space in a spheroid). A machine learning image segmenting approach revealed significantly increased extracellular space in OXT-treated spheroids. Normal distribution tested by Kolmogorov-Smirnov and Shapiro-Wilk test, two-tailed, unpaired t-test p < 0.0001, t = 5,365, n(control) = 15, n(OXT) = 35. [file Image_2.tif]

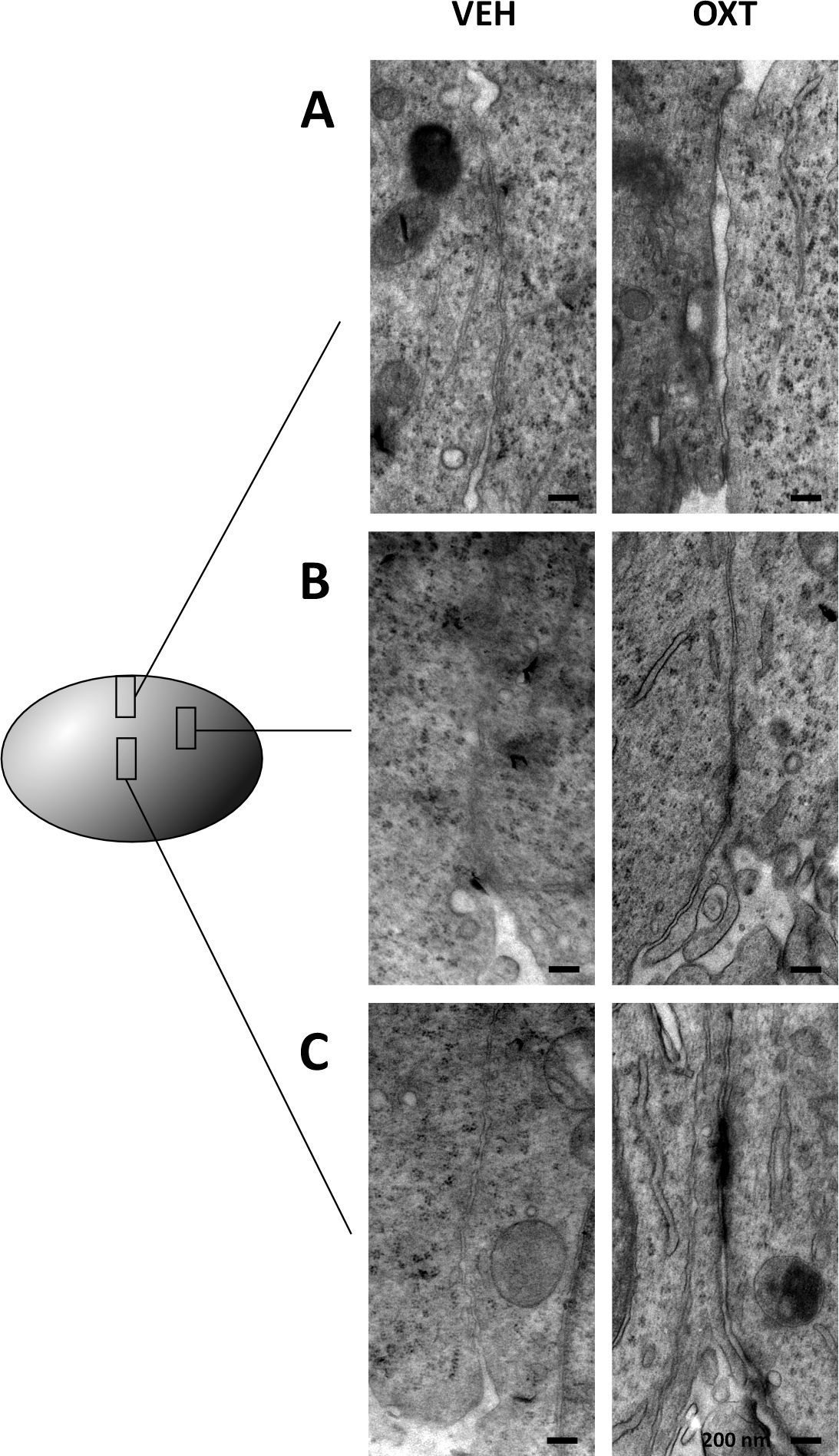

Supplement: Supplementary Figure 3 — Evaluation of spatial distribution of tight junction formation. The OXT-induced increase in extracellular space and tight junction formation is independent of the position within the spheroid. Tight junctions (OXT) or cell-cell borders (controls) are similar at the center, near center, or periphery of the spheroid. [file Image_3.jpeg]
